# Tobacco Exposure by Various Modes May Alter Proinflammatory (IL-12) and Anti-Inflammatory (IL-10) Levels and Affects the Survival of Prostate Carcinoma Patients: An Explorative Study in North Indian Population

**DOI:** 10.1155/2014/158530

**Published:** 2014-08-07

**Authors:** Shailendra Dwivedi, Apul Goel, Sanjay Khattri, Anil Mandhani, Praveen Sharma, Kamlesh Kumar Pant

**Affiliations:** ^1^Department of Biochemistry, All India Institute of Medical Sciences (AIIMS), Jodhpur, Rajasthan 342005, India; ^2^Department of Urology, King George's Medical University (KGMU), Lucknow, Uttar Pradesh 226003, India; ^3^Department of Pharmacology and Therapeutics, King George's Medical University (KGMU), Lucknow, Uttar Pradesh 226003, India; ^4^Department of Urology, Sanjay Gandhi Post Graduate Institute of Medical Sciences, Lucknow 226014, India

## Abstract

*Objective*. Inflammation is an important hallmark of all cancers and net inflammatory response is determined by a delicate balance between pro- and anti-inflammatory cytokines, which may be affected by tobacco exposure, so the present study was designed to explore the effect of various modes of tobacco exposure on interleukin-12 (IL-12) and interleukin-10 (IL-10) inflammatory cytokine levels and survival in prostate carcinoma (PCa) patients. *Methods*. 285 cancer patients and equal controls with 94 BPH (benign prostatic hyperplasia) were recruited; baseline levels of serum IL-12 and IL-10 were measured and analyzed in various tobacco exposed groups by appropriate statistical tool. Five-year survivals of patients were analyzed by Log-rank (Mantel-Cox) test (graph pad version 5). *Results*. The expression of serum proinflammatory (IL-12) and anti-inflammatory (IL-10) cytokines was correlated with tobacco exposed group as smokers, chewers, and alcohol users have shown significantly higher levels (*P* < 0.001) with significantly lower median survivals (27.1 months, standard error = 2.86, and 95% CI: 21.4–32.62); than nonusers. Stages III and IV of tobacco addicted patients have also shown significantly increased levels of IL-12 and IL-10. *Conclusions*. IL-12 and IL-10 seem to be affected by various modes of tobacco exposure and inflammation also affects median survival of cancer patients.

## 1. Introduction

Globally, prostate cancer (PCa) is the second most commonly diagnosed cancer in men (13.6% of the total) with the fifth most common cancer overall and sixth leading cause of cancer death in men [1]. In India, the age standardized incidence rates (per 10.5) of prostate cancer vary between Delhi (11.5), Mumbai (6.3), Chennai (5.2), Bangalore (6.0), and Barshi (1.6) [2]. The paradox of carcinogenesis has been now simplified into few hallmarks by unravelling the gene-environment interactions. Important hallmarks included so far are sustaining proliferative signalling, evading growth suppressors, resisting cell death, enabling replicative immortality, inducing angiogenesis, and activating invasion and metastasis [3]. Inflammation and cancer has also been linked since 1863, when Rudolph Virchow explored leucocytes in neoplastic tissue [4]. Recent studies have begun to decipher molecular pathways linking inflammation and cancer. It has been now recognized that inflammation contributes to proliferation, malignancy, angiogenesis, metastasis, adaptive immunity modulation, and unresponsiveness to hormones and chemotherapeutic agents [5]. Inflammation has been correlated with various cancers such as lung, gastric, pancreatic [6–8], and prostate cancer [9–11]. Chronic inflammation has been concerned as an important environmental influence that can cause cancer. Recent study showed that the cause of chronic inflammation in cancer patients was chronic infection in 20%, tobacco exposure and inhaled pollutant in 30%, and dietary factors in 35% [12]. Inflammation has also been linked in various steps including tumorigenesis, cellular transformation, survival, proliferation, invasion, angiogenesis, and metastasis [13].

Tobacco consumption has been correlated with various human cancers including lung, oral cavity, breast, esophagus, pharynx, larynx, and urinary bladder cancers [14–17], but its association with prostate carcinoma is controversial [18, 19]. Among the identified environmental risk factors for cancers, tobacco exposure is the leading preventable risk factor [20]. The habit of smoking and betel quid chewing is most frequent in many Asian countries including India [21]. Previously our group has reported link between IL-18 (proinflammatory) and tobacco consumption in PCa, but overall inflammation status due to anti-inflammatory levels was not explored [10]. As inflammation eventually affects the immune system of body, chronic inflammation may increase the risk of development and progression of PCa as compared to those without inflammation.

Tobacco consumption was shown to be the most important single risk factor for cancer, accounting for an estimated 20% of all cancer deaths worldwide [22]. Several studies have shown elevated levels of proinflammatory cytokines IL (IL-6, IL-15, IL-17, and IL-18) in various malignancies [9, 23–26].

Interleukin-12 (IL-12) is a pleiotropic cytokine produced primarily by monocytes and natural killer (NK) cells, dendritic cells, Langerhans cells, keratinocytes, and Kupffer cells. Interleukin-12 (IL-12) is recognized as a master regulator of adaptive type 1, cell-mediated immunity, the decisive pathway involved in protection against neoplasia and many viruses. This is supported by the analysis of animal [27] and human clinical studies that affects clinical outcome [28]. This interleukin stimulates Th1 lymphocytes development, proliferation, and cytokine production. It has been shown to trigger cytotoxicity in inflammatory diseases and against development of many kinds of malignant tumors. The effect of IL-12 on tumors is mediated by NK cells, helper cells, and cytotoxic T cells and is associated with interferon-*γ* (IFN-*γ*) production [29, 30].

Interleukin-10 (IL-10) an anti-inflammatory cytokine produced by Th2 cells, was originally termed as cytokine synthesis inhibitory factor [31], as it has a role in inhibiting cytokine production of Th1 cells. Meanwhile studies showed that the actions of IL-10 on inhibition of proinflammatory cytokine production by both T and NK cells were indirect, acting via inhibition of accessory cell function [32, 33]. In vitro and in vivo studies revealed pleiotropic activities of IL-10 on B and T cells and taken together, that a critical function of IL-10 is to suppress multiple immune responses through individual actions on T cells, B cells, antigen presenting cells and other cell types and to skew the immune response from Th1 to Th2 [34]. In malignancy, this might imply a priori that IL-10 might promote tumour development, by acting to suppress anti-tumour immune responses.

Overall inflammation status in tobacco exposed prostate cancer, control, and BPH (benign prostatic hyperplasia) is still unclear. As BPH has almost identical initial symptoms and increased PSA with enlargement of prostate glands with a low to high grade of inflammation in BPH [35, 36], in view of this, we also included BPH as one of the study groups for comparison with other groups. Thus current study was designed to explore the association of various modes of tobacco exposure with proinflammatory (IL-12) and anti-inflammatory (IL-10) levels and survival in prostate carcinoma patients.

## 2. Materials and Methods

### 2.1. Patient and Control Selection

All newly diagnosed, previously untreated 285 men with PCa attending urologic clinics of Sanjay Gandhi Post Graduate Institute of Medical Sciences, Lucknow and King George's Medical University (Earlier CSMMU), Lucknow, India, between 2007 and 2013 were included in the study. During the same period, age-matched 285 independent (of patients) healthy subjects as controls and 94 patients of BPH were recruited for comparison. All controls were free from personal or family history of cancer or any other serious illnesses, enrolled by organizing various camps. The patients and controls suffering from diabetes, arthritis, cardiovascular disease, hepatitis, AIDS, and other inflammatory diseases including prostatitis were excluded. All the subjects were above 40 years and below 80 years in age.

The ethical clearance was received from the Institutional Ethics committee. Following an informed consent, information was obtained from the subjects as age, gender, habitual attributes (recall basis), and family history for any cancer. All study subjects completed a questionnaire covering medical, residential, and occupational history. All newly diagnosed biopsy approved (pathological test) men with PCa were recruited and sampling was done before their treatment. To rule out the effect of hormone or medicine, no specific standard medicine/hormonal therapy was given before sampling; after confirmed biopsy diagnosis, men with prostate carcinoma were suggested treatment according to disease status. 5 mL blood samples (base line) of all subjects were taken at the time of admission.

### 2.2. Serum Separation and ELISA

Immediately after blood sampling, serum was obtained by centrifugation at 2000 r/min for 15 min at 4°C and stored at −80°C until later analysis. Serum PSA, IL-12 (Bender Med Systems, ELISA kits Vienna, Austria), and IL-10 (R&D systems ELISA kits) levels were determined using ELISA kits as per standard protocol of manufacturers.

### 2.3. Environmental Factors

#### 2.3.1. Exposure Factors

The exposure factors were recorded in cases (PCa and BPH) and controls, which included tobacco use (smoking and chewing tobacco) and alcohol intake. Tobacco habit was categorized into smokers and chewers (use of non-moking tobacco as powder or in beetle leaf or areca nut, catechu) and nonusers as those who were not smoking, chewing, and drinking. Smokers were defined as those who have been smoking for the last ten years or more in the form of cigarettes or bidis (hand-manufactured cigarettes consisting of tobacco wrapped in a tendu or temburini leaf) or any other smoked form as hookah (Indian water pipe), chillum, or any other smoked form not less than 20 times weekly for the last 10 years or more. Similarly, tobacco chewers were defined as those who have been using more than 20 packets of chewable tobacco products weekly: Khaini (tobacco-lime mixtures), gutkha (tobacco with betel nut, catechu, lime, and flavorings), or betel quid (zarda paan) with tobacco for last 10 years or more. Alcohol drinkers were defined as those who consumed any alcoholic beverages (e.g., beer, wine, and spirits) not less than 750 mL/week for 10 years or more. Moreover we also subgrouped the various combinations of exposure as smokers with alcohol, chewers with alcohol, combination of the above two exposures with alcohol, and alcohol alone. Information was gathered on the age of initiation of smoking and the self-reported quantity of specific tobacco products consumed by the users. Special emphasis was laid on the form of tobacco, which was used by the subjects, and the duration of consumption was noted. All modes of tobacco exposed subjects (as tobacco chewing or smoking and drinking alcohol) were considered for the study, which had the exposure history of more than 10 years.

### 2.4. Statistical Analysis

Data were summarized as mean ± SD and in percentages. Initially the analysis of variance ANOVA (one-way and two-way) was applied among various goups of tobacco users, nonusers subjects of control, cancerous patients and BPH group, if found statistically significant among groups, then pair wise comparison was performed between groups by using independent unpaired *t*-test. All the analysis was carried out by using SPSS 15.0 and Graph Pad Prism (version 5.0). The *P* value < 0.05 was considered as statistically significant.

### 2.5. Survival

The survival time of a cancer patient is defined as the time that elapsed between diagnosis and death and further their survival was studied prospectively. This requires giving a weight between 0 and 1 to the months of life lived between diagnosis and death, to reflect the quality of these life-years (where 0 = perfect health and 1 = dead). The most basic measure of patient survival is the observed survival, with the monthly for 4 years and nine months, observed survival being the percentage of patients alive after 5 years of followup from the date of diagnosis.

## 3. Results

### 3.1. Distribution of Patients by Habit of Addiction

The habit of betel quid was higher among 35.7% of BPH patients than PCa (27.8%) and controls (22.5%). However, 35.7% of BPH, 48.6% of cancer, and 40.8% of controls were using Gutkha. The habit of khaini was higher among control (36.6%) patients than BPH (28.6%) and cancer (23.6%); other habitual attributes are summarized in Table 1. The habit of tobacco smoking was also almost similar among all the groups.

Two-way analysis of variance (ANOVA) results showed significant effect between independent variables of all modes of tobacco exposure with categorical group (controls, BPH, and PCa) of subject. IL-10 levels in BPH, PCa, and control groups with various mode of exposure showed a significant effect in interaction of the data with row and column factor (with interaction of 2.42% of total variance, column factor 87.77%, and row factor 3.14%). Similarly IL-12 levels when compared in all groups with the same exposure it was again significant (with interaction of 0.76% of total variance, column factor 96.97 and row factor 1.36%). PSA levels in all three groups with all modes tobacco exposure showed significant influence only in column factor (58.90% of total variation), while interaction variation and row factor variation (0.16% and 0.09%, resp.) were insignificant.

### 3.2. Survival Analysis in PCa with Various Mode of Tobacco Exposure

**Figure 1:**
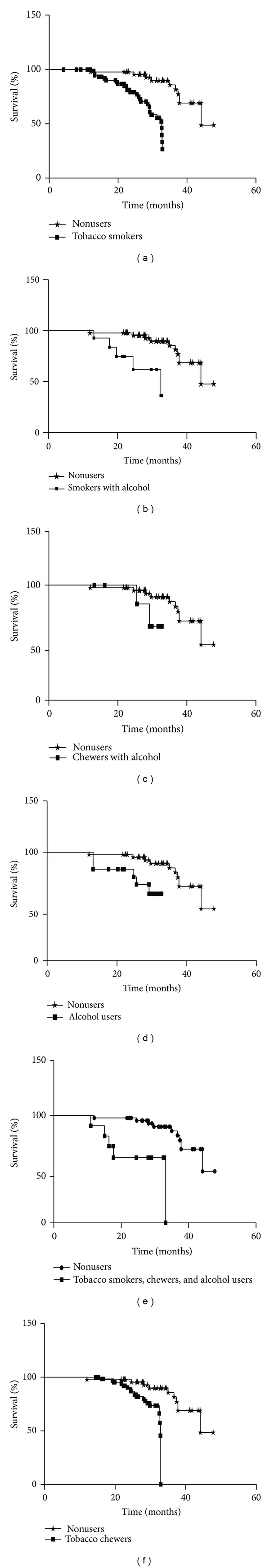
The percent survival outcome for the prostate carcinoma patients with different modes of tobacco exposed subgroup versus nonusers shown in above figures: the vertical line (*y*-axis) represents percent survival and horizontal line (*x*-axis) represents time in months. Survival graph of various subgroups included in figure are (a) tobacco smokers versus nonusers, (b) smokers with alcohol versus nonusers, (c) chewers with alcohol versus nonusers, (d) alcohol users versus nonusers, (e) smokers, chewers, and alcoholic users versus nonusers, and (f) tobacco chewers versus nonusers. All tobacco exposed subgroups of PCa patients have significantly (*P* < 0.05) lower survival rates than nonusers (except chewer with alcohol).

The median survival of nonusers was better than all tobacco exposed subgroups (43.03 months; 95% CI = 40.34–45.72). The median survival for various modes of tobacco exposed subgroups versus nonusers has been shown in Table 5 and Figures 1(a), 1(b), 1(c), 1(d), 1(e), and 1(f). The median survival in various subgroups was observed as (a) tobacco smokers (29.17 months; 95% CI = 27.80–30.54, *P* < 0.0001), (b) smoker with alcohol (28.40 months; 95% CI = 24.79–45.72, *P* = 0.001), (c) chewers with alcohol (30.16 months; 95% CI = 29.12–33.20, *P* > 0.05), and (d) with alcohol (28.90 months; 95% CI = 26.01–31.79, *P* = 0.01), while in (e) smokers, chewers, and alcoholic users (27.01 months; 95% CI = 21.40–32.62, *P* < 0.0001) and (f) tobacco chewers it was 30.93 months (95% CI = 29.99–31.88) which was lower than the nonusers (nonsmokers + nonchewers + nonalcoholic cancer patients); this difference was statistically significant (*P* < 0.0001).

### 3.3. Proinflammatory (IL-12) and Anti-Inflammatory (IL-10) Trends with Tobacco Exposure

IL-12, IL-10, and PSA levels of all tobacco exposure groups (smokers, chewers alone, and in combinations,) of control, BPH, and cancer groups were summarized in Table 2.

#### 3.3.1. Tobacco Smokers

Current result showed that the IL- 12 levels in tobacco smokers (between groups) as cigarette, bidi's, hookah and chillum users of cancer group were higher the than BPH and control group. Further when levels were compared with nonusers within the same group then all nonusers have their lowest levels. Further, among tobacco exposure groups (within groups), the mean level of IL-12 showed unique trend; that is, the levels were highest in bidi's smokers, followed by chillum, cigarette, and hookah (bidi's smokers > chillum > cigarette smokers > Hookah).

Within controls IL-12 levels differed significantly (*P* < 0.05) with tobacco smoking (by all modes i.e., overall) in bidi's and chillum users, when compared with nonusers, while it was not significant (*P* > 0.05) with cigarette.

When we compared within cancer group's IL-12 levels differed significantly (*P* < 0.05) with cigarette, bidi, hookah, chillum than nonusers cancer patients. On comparing the IL-12 levels between cancer patients and controls within same mode exposure groups, tobacco smoking (overall) showed significant difference (*P* < 0.0001).

Anti-inflammatory cytokine (IL-10) levels were found to be significantly increased (*P* < 0.05) in tobacco smokers (except hookah smokers) of PCa group than in BPH and controls. Further when compared within same group it was significantly higher in bidi, cigarette, and chillum in cancer and control groups and insignificant in BPH groups.

#### 3.3.2. Tobacco Chewers

Table 2 showed that the IL-12 levels in tobacco chewers (between groups) as betel quid, gutkha, and khaini chewers of cancer have elevated levels than BPH and control group. Moreover, when comparison was made within same group again nonusers had the lowest expressions. Further, among tobacco exposure groups (within groups), the mean level of IL-12 showed a specific trend; that is, the levels were highest in khaini chewers, followed by gutkha and betel quid chewers (khaini > gutkha > betel quid chewers).

Within controls, IL-12 levels differed significantly (*P* < 0.05) with tobacco chewers (by all modes i.e., overall) of khaini, gutkha, and betel quid chewers as compared with nonusers. On comparing the IL-12 levels among cancer patients, BPH, and controls within same mode of exposure, tobacco chewing (overall), gutkha and khaini chewers showed significant difference (*P* < 0.01) than tobacco chewers of BPH and controls and when compared within same group it showed unique trends as betel quid chewers showed significant (*P* < 0.05) higher levels in all three groups.

#### 3.3.3. Tobacco Exposure in Combination

Table 2 summarized the interleukin-12 levels in all three groups of cancer, BPH, and control and it showed that combined tobacco users (smokers with alcohols, chewers with alcohol, and chewers and smokers with alcohol) have shown significant (*P* < 0.001) increased levels of IL-12 than control and nonusers of the same exposure group. Further, among tobacco exposure in combined groups (within groups), the mean level of IL-12 showed unique trend; that is, the levels were highest in chewing and smoking with alcohol (CSA), followed by chewing with alcohol, smoking with alcohol, and alcohol alone (chewers and smokers with alcohol (CSA) > chewers with alcohol > smokers with alcohol > alcohol alone).

Within controls and BPH group, IL-12 levels differed significantly (*P* < 0.05) and showed the same trends as in cancer group. On comparing the IL-12 levels among cancer, BPH and controls within same mode of exposure of chewers and smokers with alcohol (CSA) and smokers with alcohol showed significant difference (*P* < 0.001) than controls. IL-10 levels were also shown significantly increased in all combined users, (except alcohol alone) of prostate cancer group. But IL-10 levels in BPH groups were not significantly differed, when compared with controls.

### 3.4. PSA Levels within and between Groups of Cancer, BPH, and Controls

Current study also evaluated the PSA levels (serum marker used in diagnosis of PCa and BPH) in all tobacco exposure strata and it had shown significant difference between all three groups of cancer, BPH, and controls; however, these differences were not significant within same group.

### 3.5. Tobacco Exposure and Inflammatory Status with Various Stages of Cancer

The IL-12 levels were significantly higher (*P* < 0.05) in men who were chewers, smokers, and alcoholic users (combined users) as compared to nonusers; moreover, the levels for stages III and IV were significantly higher in all modes except in tobacco chewers of stage III patients, although the levels were higher than stage II. The IL-12 levels were higher in men who were chewers and smokers as compared to nonusers in stages I and II; the results were presented in Table 3. IL-10 levels that were summarized in Table 4 have shown their increased levels in all higher stages and within all groups and they were significantly differed (*P* < 0.05) in stages II, III, and IV within all exposure groups except in nonusers and chewers with alcohol group.

## 4. Discussion

Present study was conducted in densely populated North Indian region to explore the association of inflammation with various modes of tobacco exposure in prostate carcinoma patients and their survivals by measuring the serum IL-12 (proinflammatory) and IL-10 (anti-inflammatory) cytokine levels. Further inflammation status was also compared with BPH patients and controls. Exposure of diverse exogenous agents as chemicals in working milieu for a long time may affect the physiological and biochemical metabolism. It may influence the prostate gland; the principal that such chemicals can amend the enzymatic activity has been recognized [37, 38]. Furthermore, animal studies confirmed that prostate tumors can be induced by administration of chemicals [39]. Several studies demonstrated that various exogenous chemicals may influence hormone levels which may in turn affect estrogen levels and androgenic stimulation of the prostate [40, 41]. Present study provides support that tobacco chewing and smoking may be important contributors for inflammation as pr-inflammatory IL-12 levels were increased in PCa patients but surprisingly we noticed that IL-10 cytokines, that is, anti-inflammatory levels, were also increased. The patients who were involved in tobacco smoking alone and smoking in combination with other modes as chewing and alcohol drinking showed significantly more increase in inflammation in carcinoma patients than nonusers, although this trend was also observed in BPH and controls. Current trend indicates that tobacco exposure for long time as noticed in addicted peoples (more than 10 years) may direct change in immune systems functioning due to inflammation and that may be the cause of their lower survival. Proinflammatory cytokines are essential factors in the recruitment and activation of inflammatory cells. The aggravation and stimulation of these proinflammatory mediators are most likely due to the activation of redox-sensitive transcription factor NF-*κ*B [42]. This transcription factor has been shown to be triggered by a wide array of agents including stress, cigarette smoke, viruses, bacteria, inflammatory stimuli, cytokines, and free radicals [43]. Tobacco smoke is a heterogeneous mixture that contains approximately 4,000 chemical compounds, including 40 substances categorized as carcinogenic to humans or animals [44]. Indices of increased local and systemic oxidative stress have been reported in cigarette smokers. Several studies proved that both the gas and particulate fractions of cigarette smoke are affluent sources of radicals [40]. A few studies reported that hookah is not harmful [45, 46] because of the conviction that the smoke gets filtered in the water [47]. Recently synergic effect has been shown by cigarette smoking, alcoholic consumption, and betel quid chewing in carcinogenesis [48], so this may be reason for highest levels of IL-12 (proinflammation) in combined users as all these exposures may have given a cumulative effect. Additionally, in bidi smokers, the IL-12 levels were also more significant than nonusers and cigarette users, as bidis contain tobacco with chemicals like hydrogen cyanide and ammonia. Bidis deliver more nicotine and contains more N-nitrosonornicotine (NNN) and 4-(methylnitrosamino)-1-(3-pyridyl)-1-butanone (NNK) in comparison to cigarettes. Moreover, compared to cigarettes, the mainstream smoke of bidi contains a higher amount of numerous toxic and mutagenic substances, including hydrogen cyanide, carbon monoxide, volatile phenols, and carcinogenic hydrocarbons such as benz[a]anthracene and benzo[a]pyrene in greater amounts than found in normal cigarettes [49]. Bidis could in fact be worse than cigarettes due to many reasons. As to keep bidis lit, smokers have to take more recurrent and deeper puffs as compared to cigarettes. So, smokers may inhale more smoke and take it deeper into lungs. Tobacco smoke contains 1,014–1,016 free radicals/puff, which include reactive aldehydes, quinines, and benzo(a)pyrene [50]. Many of these are comparatively long lasting, such as tar-semiquinone. ROS has been concerned in initiating inflammatory responses in the lungs through the activation of transcription factors, such as nuclear factor NF-*κ*B and activator protein- (AP-) 1 and other signal transduction pathways, such as mitogen-activated protein (MAP) kinases and phosphoinositide-3-kinase (PI-3K), leading to enhanced gene expression of proinflammatory mediators [51, 52]. Moreover, it has been revealed that oxidative stress and the redox status of the cells can also control nuclear histone modifications, such as acetylation, methylation, and phosphorylation, leading to chromatin remodelling and recruitment of basal transcription factors and RNA polymerase II leading to the induction of proinflammatory mediators [51]. Among these, NF-*κ*B has been reported to play a vital role in mediating cell survival and the upregulation of many cytokines and proinflammatory mediators essential to the host and ERK1/2 has been reported to mediate transcription of proteases and cytokines in response to an array of stimuli, including cigarette smokers [52]. In experimental systems, exposure to chewable tobacco products was linked with the generation of reactive oxygen species, modulation of inflammatory mediators, and inhibition of collagen synthesis and impairment of DNA repair capacity [53]. This study also showed unique trends of IL-12 expression in subjects of betel chewers alone and betel chewers with alcohol drink. Chewers with alcohol users of PCa patients have shown slightly higher inflammatory levels but less than other chewers and smokers as compared to nonusers. This could be because of the antioxidant and anti-inflammatory properties of betel [54].

Likewise, areca nut or seed was found to be taken simultaneously with betel and gutkha chewing which also has a strong antioxidant activity [55], but slightly higher IL-12 levels may be due to tobacco use with it. Present study indicates the ability of betel leaf to downregulate T-helper 1 proinflammatory responses [56]. Additionally, the difference in interleukin-12 levels in various modes may be due to processed and unprocessed tobacco or its products in its chewing form and smoking form. Our study does not prove that tobacco is an etiological factor for cancer prostate. Our study, however, shows overall status of inflammation by examining IL-12 (proinflammatory) and IL-10 (anti-inflammatory) level in men with PCa, BPH, and healthy controls who were smokers, gutkha users, and combined tobacco and alcohol users. In our previous study, it was shown that IL-18 levels were associated with disease progression (TNM staging) and also elevated in tobacco exposed patients of carcinoma [9, 10].

The increase in IL-10 with IL-12 seems functionally quite dissimilar as IL-10 is produced by Th2 cells and inhibits cytokine production of Th1 cells (IL-12). However studies showed that the actions of IL-10 on inhibition of proinflammatory cytokine production by both T and NK cells were indirect, acting via inhibition of accessory cell function [32, 33]. So this increase may be due to inhibition of the Th1 (IL-12) cytokine production and to promotion of tumor progression by suppressing antitumor response [57], which ultimately may lead to poor survival. But antagonistically IL-10 (Th2) aggravates the suppression of IL-12 (Th1) induced proinflammation and eventually it mounts poor immune response.

The combined users (chewers, smokers, and alcohol) have higher proinflammatory levels and consequently aggravated anti-inflammatory response (IL-10) that may be a reason for their poor survival. The role of inflammation and immunity in tumor biology is complex. When the immune response is functioning normally, inflammation is self-limiting. The production of proinflammatory or Th-1 cytokines is followed by anti-inflammatory or Th-2 cytokines [58]. Furthermore our report is also inconsistent with other cancer studies that reported a higher IL-12, IL-10 levels with worse survival [59, 60].

This study may become more vital if larger sample size in all strata of tobacco exposure, namely, betel quid, hookah, chillum, and so forth, were included, as current sample size and finding is from our prospective data of our study on prostate cancer patients, who come for treatment during study time. Strongest point of this study is that our group tried to explore complete inflammation status by evaluating proinflammatory marker (IL-12) and anti-inflammatory (IL-10) levels in tobacco addicted groups and its impact on survivals. This type of study will definitely solve the paradox of tobacco exposure in development of various untreated disease like cancers.

## 5. Conclusions

Tobacco exposure has always been characterized as main attributable risk factor in the development of various cancers; this study helps to understand the link between tobacco exposure-mediated inflammatory response and PCa development/progression. Present study examined the effect of tobacco exposure in different modes of smoking, chewing alone, and in combination with alcohol and observed increased IL-12 (proinflammatory) with IL-10 (anti-inflammatory) levels in comparison to nonusers of the same group of PCa, BPH. Further it has also been observed that patients with prostate carcinoma exposed to smoking and chewing with alcohol has poor survival due to elevation in IL-10 levels which suppresses immune response mounted by increased IL-12 against cancer. Thus study concludes that quitting of tobacco abuse may provide protection against progression of cancer and improves survival.

## Figures and Tables

**Table 1:** Distribution of patients by habit of addiction.

Addiction	Groups					
	BPH (*n* = 94)		Cancer (*n* = 285)		Controls (*n* = 285)	
	Number	%	Number	%	Number	%
Tobacco chewing	*n * *** *** *= * *** *28**	**29.7**	*n * *** *** *= * *** *72**	**25.3**	*n * *** *** *= * *** *71**	**24.9**
Betel quid (leaf + areca catechu with tobacco)	10	**10.6** (35.7)	20	**7.0** (27.8)	16	**5.6** (22.5)
Gutkha	10	**10.6** (35.7)	35	**12.3** (48.6)	29	**10.2** (40.8)
Khaini	8	** 8.5** (28.6)	17	**5.9** (23.6)	26	**9.1** (36.6)
Tobacco smoking	*n * *** *** *= * *** *31**	**32.97**	*n * *** *** *= * *** *92**	**32.3**	*n * *** *** *= * *** *88**	**30.9**
Cigarette	11	**11.7** (35.5)	43	**15.1** (46.7)	40	**14** (45.5)
Bidi	11	**11.7** (35.5)	31	**10.9** (33.7)	30	**10.5** (34.1)
Hookah	4	**4.2** (12.9)	11	**3.8** (12.0)	11	** 3.8** (12.5)
Chillum	5	**5.3** (16.1)	7	**2.4** (7.6)	7	**2.4** (8.0)
Smoking with alcohol	**11**	**11.7**	**34**	**11.9**	**34**	**11.9**
Chewing with alcohol	**6**	**6.4**	**19**	**6.7**	**13**	**4.6**
Alcohol alone	**9**	**9.6**	**16**	**5.6**	**14**	**4.9**
Smoking, chewing, and alcohol using	**5**	**5.3**	**10**	**3.5**	**6**	**2.1**
Nonusers	**4**	**4.3**	**42**	**14.7**	**59**	**20.7**

**Table 2:** Tobacco and alcohol exposure with various modes and their association with pro-inflammatory (IL-12) and anti-inflammatory (IL-10) cytokine and PSA in all groups.

Addiction	Interleukin-12 (pg/mL)			Interleukin-10 (pg/mL)			PSA (ng/mL)		
	BPH (n = 94)	Cancer (n = 285)	Controls (n = 285)	BPH (n = 94)	Cancer (n = 285)	Controls (n = 285)	BPH (n = 94)	Cancer (n = 285)	Controls (n = 285)
Tobacco chewing									
Betel quid	65.69 ± 2.84^ns^	164.64 ± 6.27∗	54.39 ± 2.55∗	3.56 ± 0.57∗	13.41 ± 1.90∗∗	3.30 ± 0.36∗∗	2.36 ± 0.20^ns^	29.72 ± 18.22^ns^	0.79 ± 0.28^ns^
Khaini	68.35 ± 3.25∗	177.91 ± 7.53∗∗	57.13 ± 2.46∗∗	2.29 ± 0.21^ns^	10.09 ± 0.46∗∗	2.60 ± 0.38∗∗	2.42 ± 0.28^ns^	28.26 ± 12.15^ns^	0.63 ± 0.23^ns^
Gutkha	66.79 ± 3.74^ns^	171.91 ± 5.47∗∗	55.20 ± 1.59∗∗	2.62 ± 0.32^ns^	9.59 ± 0.70∗	2.59 ± 0.31∗∗	2.31 ± 0.32^ns^	25.99 ± 12.08^ns^	0.66 ± 0.23^ns^
Tobacco smoking									
Cigarette	65.57 ± 2.64^ns^	167.58 ± 5.87∗∗	53.11 ± 2.33^ns^	2.36 ± 0.17^ns^	9.21 ± 0.74∗	2.68 ± 0.33∗∗	2.23 ± 0.14^ns^	26.83 ± 12.60^ns^	0.58 ± 0.25^ns^
Bidi	68.97 ± 2.25∗	183.81 ± 8.64∗∗	57.25 ± 4.08∗∗	2.63 ± 0.22^ns^	9.59 ± 0.73∗∗	2.86 ± 0.25∗∗	2.28 ± 0.18^ns^	27.01 ± 9.76^ns^	0.62 ± 0.31^ns^
Hookah	63.59 ± 1.13^ns^	163.50 ± 5.39∗∗	53.33 ± 1.90^ns^	2.60 ± 0.27^ns^	8.97 ± 0.78^ns^	2.58 ± 0.29^ns^	2.24 ± 0.15^ns^	28.63 ± 17.08^ns^	0.67 ± 0.35^ns^
Chillum	68.66 ± 3.47^ns^	183.81 ± 3.97∗∗	56.79 ± 2.50∗∗	2.64 ± 0.25^ns^	9.92 ± 0.64∗	2.74 ± 0.30∗	2.34 ± 0.20^ns^	27.22 ± 12.91^ns^	0.92 ± 0.68^ns^
Smoking with alcohol	70.03 ± 3.83∗∗	189.56 ± 6.27∗∗	58.09 ± 2.55∗∗	2.39 ± 1.62^ns^	11.70 ± 1.16∗∗	2.97 ± 0.28∗∗	2.35 ± 0.27^ns^	26.87 ± 9.41^ns^	0.68 ± 0.27^ns^
Chewing with alcohol	72.38 ± 3.16∗∗	194.90 ± 6.03∗∗	59.32 ± 2.58∗∗	2.62 ± 0.19^ns^	11.84 ± 1.20∗∗	3.08 ± 0.33∗∗	2.47 ± 0.28^ns^	25.05 ± 10.11^ns^	0.64 ± 0.28^ns^
Alcohol alone	71.20 ± 2.63∗∗	187.25 ± 5.38∗∗	55.2 ± 1.20∗∗	2.53 ± 0.36^ns^	8.97 ± 0.76^ns^	2.76 ± 0.36∗∗	2.35 ± 0.19^ns^	25.13 ± 10.22^ns^	0.62 ± 0.36^ns^
Smoker & chewing and alcohol	74.09 ± 1.29∗∗	204.12 ± 2.78∗∗	61.11 ± 3.11∗∗	2.52 ± 0.24^ns^	11.99 ± 1.49∗∗	3.22 ± 0.27	2.38 ± 0.30^ns^	28.46 ± 12.32^ns^	0.64 ± 0.31^ns^
Non-users	62.04 ± 1.05	158.63 ± 4.32	51.46 ± 3.23	2.06 ± 0.51	8.47 ± 0.98	2.24 ± 0.31	2.27 ± 0.17	26.39 ± 11.63	0.61 ± 0.27

^ns^P > 0.05, *P < 0.05, **P < 0.001 comparison with non users within same group.

**Table 3:** Stagewise expression of proinflammatory (IL-12) cytokine with various mode of tobacco and nonusers in carcinoma prostate patients.

Stage	Tobacco chewers	Tobacco smokers	Smokers with alcohol	Chewers with alcohol	Alcohol alone	Smokers, chewers, and alcohol users	Nonusers
Stage I	166.74 ± 4.68	169.36 ± 2.81	171.14 ± 3.14	173.18 ± 4.37	169.51 ± 3.67	182.74 ± 5.48	152.12 ± 2.41
Stage II	168.37 ± 6.39^ns^	172.51 ± 3.63^ns^	174.63 ± 2.79∗	175.36 ± 2.94^ns^	173.81 ± 3.81∗∗	186.74 ± 4.87^ns^	152.63 ± 3.26^ns^
Stage III	169.21 ± 5.86^ns^	175.27 ± 3.26∗∗	175.86 ± 3.93∗∗	179.92 ± 3.23∗∗	175.36 ± 2.98∗∗	189.82 ± 4.36∗∗	154.73 ± 2.67∗
Stage IV	174.59 ± 7.54∗∗	181.67 ± 5.84∗∗	183.75 ± 2.42∗∗	183.34 ± 4.86∗∗	182.22 ± 4.23∗∗	196.74 ± 6.89∗∗	157.33 ± 3.89∗∗

^ns^ *P* > 0.05, *P < 0.05, and **P < 0.001 comparison with stage I within the same group.

**Table 4:** Stagewise expression of anti-inflammatory (IL-10) cytokine with various mode of tobacco and nonusers in carcinoma prostate patients.

Stage	Tobacco chewer	Tobacco smokers	Smokers with alcohol	Chewers with alcohol	Alcohol alone	Smokers, chewers, and alcohol users	Nonusers
Stage I	7.13 ± 0.96	7.67 ± 0.73	6.91 ± 1.03	9.84 ± 0.97	6.89 ± 0.52	7.13 ± 1.21	6.97 ± 1.32
Stage II	10.35 ± 1.21∗∗	8.74 ± 0.57∗∗	8.67 ± 0.89∗∗	10.38 ± 1.21^ns^	8.05 ± 0.73∗∗	9.69 ± 0.82∗∗	7.47 ± 0.98^ns^
Stage III	10.89 ± 0.87∗∗	8.99 ± 0.41∗∗	9.26 ± 1.21∗∗	11.06 ± 1.61∗∗	9.34 ± 0.68∗∗	10.92 ± 1.14∗∗	8.12 ± 1.05∗∗
Stage IV	12.58 ± 1.19∗∗	9.07 ± 1.04∗∗	12.39 ± 1.16∗∗	11.34 ± 0.78∗∗	9.72 ± 0.75∗∗	11.46 ± 1.32∗∗	9.40 ± 1.12∗∗

^ns^ *P* > 0.05, *P < 0.05, and **P < 0.001 comparison with stage I within the same group.

**Table 5:** Comparison of survival time (in months) among tobacco, alcohol, and nonusers.

Habit	Median survival in months			
	Estimate	Std. error	95% confidence interval	
Tobacco chewers∗	30.93	0.48	29.99	31.88
Tobacco smokers∗	29.17	0.70	27.80	30.54
Smokers with alcohol∗∗	28.40	1.84	24.79	45.72
Chewers with alcohol∗∗∗	31.16	1.04	29.12	33.20
With alcohol∗∗∗∗	28.90	1.37	26.01	31.79
Smokers, chewers, and alcohol users∗	27.01	2.86	21.40	32.62
Nonusers	43.03	1.37	40.34	45.72

log⁡rank⁡  *P value < 0.0001, **P = 0.001, ***P = 0.12, and ****P = 0.01.
